# Exploring the factors influencing the intention to clothing and textiles recycling among Chinese college students’: a study based on TPB and VBN

**DOI:** 10.3389/fpsyg.2023.1328037

**Published:** 2024-01-11

**Authors:** Ningna Sun, Dong Liu, Jianrui Zhang

**Affiliations:** Department of Industrial Design, School of Art, Jiangsu University, Zhenjiang, Jiangsu, China

**Keywords:** clothing and textiles recycling, Chinese college students, behavioral intention, theory of planned behavior, value-belief-norm theory

## Abstract

Recycling apparel and fabrics is essential for preserving resources and protecting the environment, providing considerable global advantages for ecology and society. This study sought to explore the participation of Chinese college students in the recycling of clothing and fabrics by combining the Theory of Planned Behavior (TPB), the Value-Belief-Norm theory (VBN), self-identity, school education, and perceived policy effectiveness to create a comprehensive model. A total of 1,027 valid samples were obtained through stratified sampling and random sampling, primarily sourced from Eastern China, and analyzed through Structural Equation Modeling (SEM) utilizing AMOS. The results of the research suggested the following: (1) College students’ biospheric values emerged as the most influential factor in predicting their inclination to participate in recycling behavior. (2) VBN (encompassing biospheric and altruistic values) and self-identity significantly contributed to shaping attitude and perceived behavioral control, which in turn impacted individuals’ intention to participate in recycling. (3) School education exerted a stronger moderating influence than perceived policy effectiveness on the relationship between attitude, perceived behavioral control, and intention. The comprehensive model proposed in this study demonstrated superior predictive capability in explaining college students’ willingness to participate in clothing and textiles recycling. It has been proven to be practical and effective. Lastly, schools should promote the practice of clothing and textiles recycling, cultivate good values, and utilize the power of social influence to encourage college students to participate in clothing and textiles recycling. The government should improve relevant laws and regulations, collaborate with academia, and adopt measures like incentives to create a pro-environment atmosphere.

## 1 Introduction

Since the advent of the first industrial revolution, global economies have witnessed remarkable growth. Nevertheless, this economic progress has come at the expense of resource depletion and environmental degradation. In recent years, human interventions in climate and the environment have exacerbated environmental deterioration at an alarming rate, presenting a substantial threat to the stability of the Earth’s ecosystem and the sustainable development of human society. This degradation, especially in the context of the COVID-19 pandemic, may trigger extreme weather events and further undermine agriculture, water resources, biodiversity, global health, among other areas (Savari and Khaleghi, 2023a; Savari et al., 2023a). Shahab et al. (2023) suggested that if the current pace of global non-renewable energy consumption continues without rational resource recycling, these energy sources could be depleted within a century. Additionally, the widespread disposal of waste products into nature has contributed to severe environmental pollution, as evidenced by notable incidents like the dead lakes in North America and the forest dieback in West Germany (Savari and Khaleghi, 2023b). Safeguarding a healthy environment is thus of paramount significance in realizing sustainable development for the well-being of human beings and the ecosystem.

In the fashion industry, the pursuit of fashion trends has accelerated clothing turnover. Annually, over 30 million tons of clothing are discarded globally, with a recycling rate of less than 5%, leading to substantial resource wastage and potential urban pollution concerns due to improper disposal (Rausch and Kopplin, 2021). In China, the main methods to dispose of discarded clothing are landfilling or incineration. Relative to incineration, recycling 1 ton of discarded textiles could save 10 t of carbon dioxide emissions (Zamani et al., 2015). Clothing and textiles recycling involves the reuse of no longer worn clothing through resale or donation, aiming to extend clothing lifespan and reduce resource waste and environmental impact (Joung and Park-Poaps, 2013). Due to its considerable environmental and societal benefits, recycling clothing and textiles is of pivotal significance for individuals (Harmsen et al., 2021; Savari et al., 2022). On the hand, the recycling of used clothing effectively reduces the demand for natural resources, promotes sustainable resource utilization, decelerates the pace of environmental pollution, mitigates the adverse impact of discarded clothing on soil and air, and catalyzes the development of a circular economy (Weber et al., 2023; Savari et al., 2023b). On the other hand, it plays a critical role in advancing sustainable practices in fashion and consumption, thereby cultivating more environmentally-friendly lifestyles among people (Sun et al., 2022). Despite the global promotion of clothing and textiles recycling, various factors, including consumer awareness and social environments, hinder its widespread adoption in developing countries.

In the case of China, the current clothing and textiles recycling mechanism relies primarily on recycling facilities while the public enthusiasm to engage in recycling activities is relatively low. Relevant data show that China boasts the largest student population globally, with 44.3 million institutions thus assume an essential role in encouraging environmental conservation and recycling and reuse efforts among college students (Wang, 2021). Besides, Universities and colleges, as key channels for recycling, provide a centralized and targeted approach to facilitate recycling and reuse activities (Hao, 2017). Given their age and identity, college students are more receptive to and capable of spreading the concept of clothing and textiles recycling (Wang, 2021). However, various factors influence college students’ behavior, and without proper guidance, maintaining positive recycling habits can be challenging. Hence, it is essential to explore the factors impacting Chinese college students’ inclination to participate in clothing and textiles recycling.

In recent years, the academic community has placed significant emphasis on clothing and textiles recycling. Zhang et al. (2020) carried out a survey among Chinese residents, utilizing the TAM and the TPB to examine their willingness to adopt second-hand platforms for disposing of used clothing. Mason et al. (2022) conducted a survey on 943 Generation Y individuals employing the TPB and demonstrated that only individuals with a higher environmental attitude strengthen the association between intention and behavior regarding clothing and textiles recycling. Hassan et al. (2022) explored the factors influencing clothing disposal behaviors within the demographic of young consumers in developing nations such as Malaysia, revealing that sustainable consumption behavior exerts a substantial and favorable influence on both charitable and economic disposal of used clothing. Rotimi et al. (2023) integrated the TPB with self-efficacy, ecological literacy, self-identity, and habitual recycling behavior, addressing the limitations of the TPB in elucidating recycling behavior.

While existing studies have laid a foundation for research in clothing and textiles recycling, certain limitations persist. Firstly, preceding research primarily relied on the TPB with few analyses of individual behavioral intentions from other theoretical perspectives. Most analyses only focus on psychological factors and do not account for external factors such as policies and situational influences (Bamberg et al., 2007). Secondly, although clothing and textiles recycling is a globally recognized issue, most studies have focused on young populations in other developing countries, with limited research conducted on the participation of young individuals in China. Lastly, existing research has mainly concentrated on direct and indirect effects, without investigating the moderating effects on individual behavioral intentions.

In behavioral study, the Theory of Planned Behavior (TPB) and the Value-Belief-Norm (VBN) theory both serve as theoretical foundations for assessing factors influencing individuals’ pro-environmental behavioral intentions (Woosnam et al., 2022). The TPB model emphasizes the collective implications of individual attitudes, subjective norms, and perceived behavioral control for behavior intentions, demonstrating robust explanatory power in the analysis of environmental behaviors (Zha et al., 2023). By comparison, the VBN model underscores the impact of values on individual behavioral intentions, with high effectiveness and applicability in the analysis of intentions related to environmental protection (Chen, 2020). Hence, the employment of TPB and VBN theories in this study holds considerable explanatory potential. Nevertheless, both theories have limitations: TPB primarily explains the subjective perceptions of individual motivation and behavior, but falls short in addressing the influence of individual behavioral values. VBN theory emphasizes the importance of environmental values but overlooks critical factors influencing individuals, such as attitudes (Bamberg et al., 2007). Combined together, TPB and VBN can complement each other, enhancing the explanatory and predictive power of individual behavioral intentions (Lane and Potter, 2007; Woosnam et al., 2022). Therefore, this study comprehensively examined the factors influencing college students’ intentions to participate in clothing and textiles recycling, covering three variables from TPB theory, i.e., attitude, subjective norms, and perceived behavioral control, as well as two variables from VBN theory, i.e., biospheric and altruistic values.

In addition, research shows that individuals’ behavioral intentions are closely associated with self-identity (Barbarossa et al., 2017; Neves and Oliveira, 2021). Self-identity involves an individual’s identification with their own status and values (Becerra et al., 2023). If college students link engagement in the recycling of used clothing with their personal environmental and social responsibility identities, they are more likely to demonstrate positive behavioral intentions. This variable can contribute to a deeper understanding of the motivations and internal factors of college students participating in clothing recycling, thereby providing a scientific basis for formulating targeted advocacy and educational strategies.

Individuals live in a complex social environment, and the sound operation of society requires effective policy support (Yang and Zhao, 2023). College students, as a well-educated group, have a stronger perception of policies. The variable of perceived policy effectiveness reflects college students’ trust and recognition of the implementation of clothing and textiles recycling policies (Oreg and Katz-Gerro, 2006; Biscaia et al., 2021). If college students believe that policies are effective and have a positive social impact, these policies will directly shape their attitudes and decision-making processes regarding recycling activities. Conversely, if college students perceive policies as ineffective, the opposite effect will occur. Therefore, the perceived effectiveness of policies is particularly important in the study of college students’ participation in clothing and textiles recycling. Similarly, college students are regarded as future champions and advocates of an environmentally friendly society, and universities, as crucial cultivators of students’ values and thoughts, play an important role in this process (Yuan and Zuo, 2013). University education can encompass the cultivation of environmental protection knowledge and a sense of social responsibility, and the quality of their environmental education directly impacts students’ attitudes and behavioral intentions toward clothing and textiles recycling (Chuvieco et al., 2022). Therefore, it is necessary to take into account school education as an important variable in the study.

In summary, this study conducted a comprehensive examination of both psychological and external factors affecting college students’ intentions to participate in clothing and textiles recycling by integrating the TPB and VBN theories and introducing three additional variables: self-identity, school education, and perceived policy effectiveness, addressing the limitations of previous studies that lacked consideration of external factors. Moreover, this study took into account the moderating effects on college students’ behavioral intentions to participate in clothing and textiles recycling. A questionnaire survey was utilized to gather data, and SPSS and AMOS were used for measurement and structural model analyses. In order to uncover key factors influencing Chinese college students’ participation in clothing and textiles recycling and provide practical suggestions, SEM was utilized to examine the hypothesized paths and causal relations among variables.

## 2 Literature review

### 2.1 The value-belief-norm theory

The VBN theory, formulated by Stern (2000), is a social psychology framework employed for elucidating individuals’ motivation and behavior in environmental actions and social participation. Within this theory, biospheric and altruistic values are significant moral drivers for fostering environmental behavior. Biospheric values emphasize respect and protection for the entire ecosystem (Ünal et al., 2019). Altruistic values are important explanatory factors for individuals’ engagement in prosocial behavior, emphasizing the prioritization of others’ interests and well-being (Kim and Seock, 2019).

The VBN theory has found extensive application in the study of environmentally friendly conduct and is considered one of the most effective theories for investigating various environmental actions (De Groot and Steg, 2009). Sanchez-Garcia et al. (2021) utilized the VBN theory to explain citizens’ inclination to provide financial support for addressing air pollution resulting from road traffic. Their findings revealed that both biospheric and altruistic values exert a substantial positive influence on behavioral intentions. Additionally, Ojea and Loureiro (2007) observed a positive correlation between participants’ altruistic values and their behavioral intentions. Similarly, Lee and Jan (2018), in their study on factors influencing ecotourism behavior among tourists, confirmed a favorable correlation between biospheric values and behavioral intentions.

In this study, biospheric values represent the extent to which college students highly value sustainability and environmental protection in their engagement in clothing and textiles recycling behavior. Altruistic values signify college students’ concern for the well-being of others and their commitment to improving societal welfare through clothing and textiles recycling. In the higher education setting, college students tend to be more concerned about the environment in respect of pollution and resource waste, and possess a moral responsibility to care for others. Consequently, their inclination to participate in clothing and textiles recycling is more prone to be activated. Considering these factors, the following research hypotheses were formulated:

*H1:* Altruistic values positively influence the intention of college students to get involved in clothing and textiles recycling.

*H2:* Biospheric values positively influence the intention of college students to participate in clothing and textiles recycling.

### 2.2 Theory of planned behavior

The extension of the Theory of Reasoned Action (TRA), the Theory of Planned Behavior (TPB), provides a theoretical framework for the analysis of individual behavior. It encompasses five components: attitude, subjective norm, perceived behavioral control, behavioral intention, and actual behavior (Ajzen and Driver, 1991). Previous research on TPB (Li et al., 2018; Choi and Johnson, 2019; Rezaei et al., 2019) has shown that behavioral intentions are influenced by three key factors: (1) attitude, which signifies the individual’s favorable or unfavorable evaluation of their likelihood to participate in a specific behavior; (2) subjective norm, which signifies the social influence recognized by the person regarding their participation in a specific behavior; (3) perceived behavioral control, which mirrors the individual’s perception of the level of ease or difficulty associated with executing a specific behavior.

TPB has been highly applicable in studying participation in environment friendly activities and behavioral intentions (Yadav et al., 2019). Several studies have confirmed that attitude stands as one of the most pivotal elements impacting behavioral intentions (Ru et al., 2019; Sanchez-Garcia et al., 2021). Zhang et al. (2020) applied the theory of TPB to investigate Chinese residents’ intention to use second-hand platforms to sell used clothing. The research revealed that behavioral intentions are impacted by both subjective norms and perceived behavioral control. Furthermore, Yadav and Pathak (2017) examined the factors influencing green purchase behavior among consumers in developing countries using TPB and found that attitude, perceived behavioral control, and subjective norm all affected behavioral intentions.

Consistency can generally be observed between college students’ attitudes and their behavioral intentions. This implies that if college students perceive clothing and textiles recycling as the right thing to do and beneficial to society, and if they support its promotion, it will encourage their active participation. In terms of subjective norms, the more support college students receive from their peers, schools, and relatives regarding their participation in clothing and textiles recycling, the more determined they are to partake in such conduct. Perceived behavioral control signifies college students’ assessment of the level of ease or difficulty associated with their engagement in clothing and textiles recycling in terms of time, effort, and available opportunities. The stronger their perception of control over participating in clothing and textiles recycling, the more robust their behavioral intention to partake in it. Considering these factors, the following research hypotheses were formulated:

*H3:* Perceived behavioral control positively influences the intention of college students to participate in clothing and textiles recycling.

*H4:* Attitude positively influences the intention of college students to engage in clothing and textiles recycling.

*H5:* Subjective norm positively influences the intention of college students to participate in clothing and textiles recycling.

### 2.3 Relationships between the TPB and VBN

This section explores the potential impact of VBN values on TPB variables and proposes corresponding research hypotheses. Firstly, the potential impact of VBN values, specifically biospheric and altruistic values, on perceived behavioral control was examined. Liebe et al. (2011) highlighted the association between individual values and perceived behavioral control, which indirectly affects participants’ behavioral intentions. Furthermore, Sanchez-Garcia et al. (2021) employed an SEM that integrated TPB and VBN, demonstrating that values can explain individual behavioral intentions through perceived behavioral control.

Secondly, the potential impact of values on attitudes was explored, with the aim of elucidating the association between values and attitudes to comprehend individual behavioral intentions. Nguyen et al. (2016) suggested that biospheric values have an indirect effect on the purchase of energy-efficient products through consumer attitudes. Additionally, Yadav et al. (2019) provided further evidence in a study on travelers’ choice of green hotels, demonstrating the positive influence of biospheric values on individual behavioral intentions and emphasizing the mediating role of attitudes in this process. Similarly, Sereenonchai and Arunrat (2021) found that altruistic values significantly influence individuals’ intention to engage in food safety behaviors through their impact on attitudes. Sanchez-Garcia et al. (2021) also reached similar conclusions, indicating that altruistic values enhance citizens’ willingness to pay for air pollution by shaping their attitudes.

In this study, college students’ positive values of sustainable development, environmental protection, social welfare, and helping others could further shape their attitudes and increase their inclination to allocate time and effort to clothing and textiles recycling. Considering these factors, the following research hypotheses were formulated:

*H6a:* Through perceived behavioral control, altruistic values have an indirect impact on the intention of college students to participate in clothes and textile recycling.

*H6b*: Through attitudes, altruistic values have an indirect impact on the intention of college students to participate in clothes and textile recycling.

*H7a*: Biospheric values indirectly influence college students’ intention to participate in clothing and textiles recycling through perceived behavioral control.

*H7b*: Biospheric values indirectly influence the intention of students in colleges to participate in clothing and textiles recycling through attitudes.

### 2.4 Self-identity, perceived policy effectiveness, and school education

Self-identity, increasingly explored in research on pro-environmental behaviors, is another significant facilitating factor for behavioral intention (Barbarossa et al., 2017; Neves and Oliveira, 2021; Rotimi et al., 2023). Self-identity measures an individual’s cognitive and acceptance level of their own traits and social status (Rex et al., 2015). Mancha and Yoder (2015) investigated individuals’ willingness to green behavior and found that self-identity is the most influential predictor of subjective norms, attitudes, and perceived behavioral control, which was reinforced by Gkargkavouzi et al. (2019).

When college students exhibit a greater degree of identification with environmental and sustainability concerns, they can be more inclined to allocate time to and exert effort in participating in clothing and textiles recycling. Consequently, their attitudes become more positive, and they are more likely to receive support from family and peers. Considering these factors, the following research hypotheses were formulated:

*H8a-c*: Self-identity has an indirect impact on college students' intention to participate in clothing and textiles reusing through perceived behavioral control, attitudes, and subjective norms, respectively.

Relevant research suggested that apart from VBN variables, perceived policy effectiveness is a significant factor in elucidating individuals’ pro-environmental behavioral intentions (Fornara et al., 2016; Lee, 2016). Within the context of the current study, perceived policy effectiveness denotes college students’ perception of policies as effective in encouraging and promoting clothing and textiles recycling (Oreg and Katz-Gerro, 2006). Wan et al. (2014a) highlighted the important moderating role of perceived policy effectiveness in influencing individuals’ behavioral intentions. When individuals perceive policies as ineffective, the impact of attitudes on behavioral intentions is weakened. Conversely, under the assumption of policy effectiveness, attitudes positively enhance individuals’ behavioral intentions. Wan et al. (2014b) further elucidated that efficacious clothing and textiles recycling serves as a substantial moderator that associates subjective norms with behavioral intentions. Conversely, diminished perceptions of policy effectiveness weaken the link between subjective norms and behavioral intentions. Additionally, Shen et al. (2022) argued that increased perceived policy effectiveness enhance the impact of perceived behavioral control on behavioral intentions. Considering these factors, the following research hypotheses were formulated:

*H9a-c*: Perceived policy effectiveness plays a positive regulating role between subjective norms, attitudes, perceived behavioral control, and college students’ intention to participate in clothing and textiles recycling, respectively.

Studies have indicated that school education is crucial in enhancing students’ understanding of sustainable development issues and fostering behavioral intentions (Diamantopoulos et al., 2003; Yuan and Zuo, 2013). Through environmental research and teaching, school can instill in college students a deeper awareness of environmental protection and concepts of sustainable development. This, in turn, promotes universities to serve as demonstration bases for clothing and textiles recycling, positively influencing and leading future societal environmental behaviors and attitudes (Clarke and Kouri, 2009; Shokati Amghani et al., 2023). Additionally, school education also bears the responsibility of cultivating future environmental leaders and policymakers, providing essential support for maintaining the normal functioning of ecosystems and safeguarding biodiversity (AlMubarak, 2023; Savari et al., 2023c). The environmental ethics and values conveyed through school education could reinforce college students’ understanding of environmental issues, foster their sense of responsibility to protect the environment and conserve resources, and encourage them to contribute to the sustainable development of society (Esteban Ibáñez et al., 2020). In the present study, school education refers to the provision of teaching, lectures, and social activities related to clothing and textiles recycling. Zhao et al. (2014) suggested that higher educational quality provided by schools leads to more positive attitudes toward the environment and stronger intentions to participate in pro-environmental behaviors among college students. Chekima et al. (2016) further demonstrated the moderating impact of school education with regard to the link between attitudes and behavioral intentions among college students, highlighting the significance of school education in shaping behavioral intentions. Additionally, a recent study on entrepreneurial intentions found that school entrepreneurship education exerts a substantial moderating influence on attitudes, subjective norms, perceived behavioral control, and entrepreneurial intentions among college students (Tseng et al., 2022).

Considering the context of clothing and textiles recycling, school education can be viewed as a crucial moderating factor between TPB variables and the intention of college students to engage in clothing and textiles recycling. Considering these factors, the following research hypotheses were formulated:

*H10a-c*: School education serves as a positive moderator in the associations between attitudes, subjective norms, perceived behavioral control, and the intention of college students to participate in clothing and textiles recycling, respectively.

**Figure 1:**
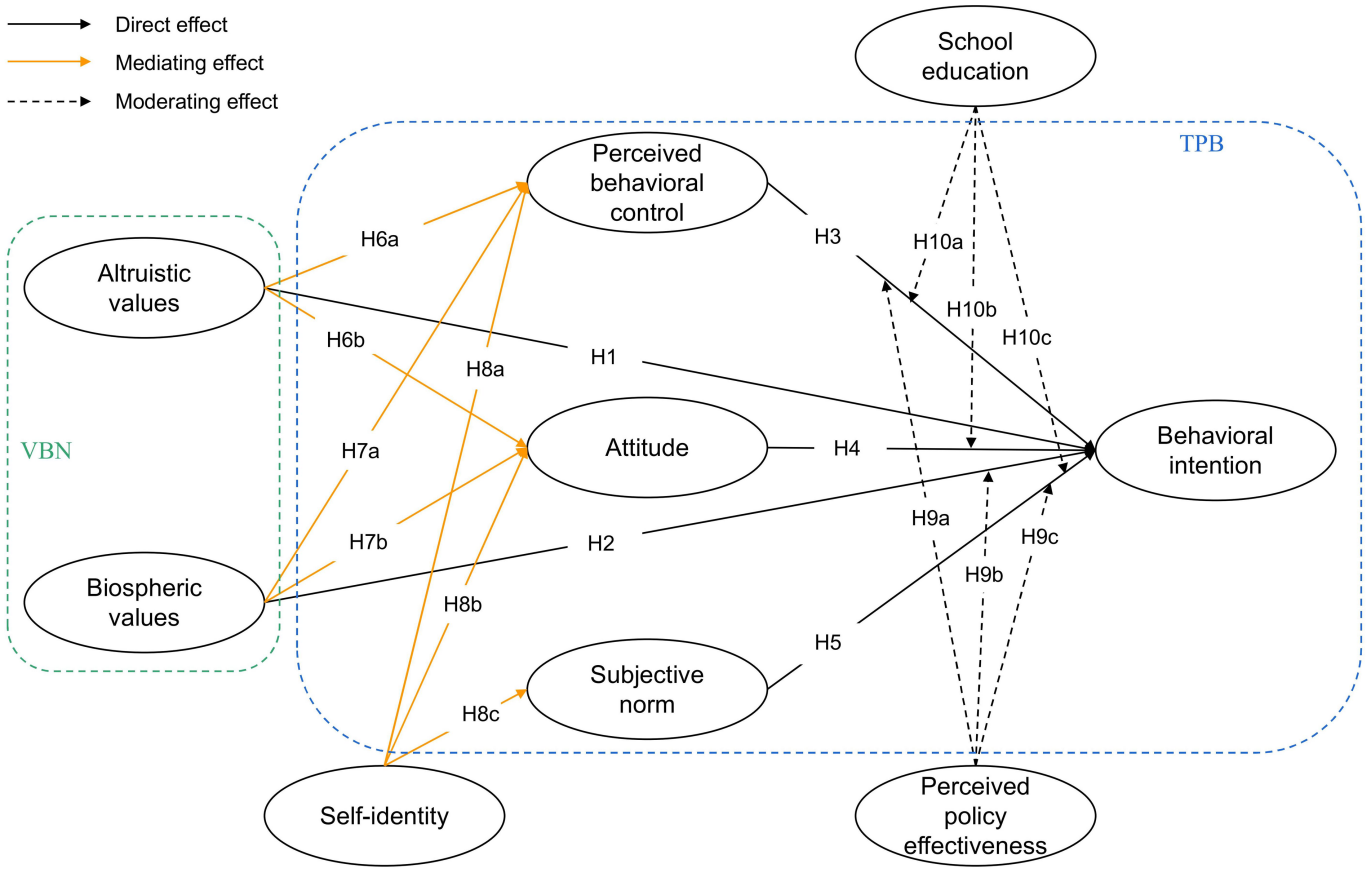
The research model.

In light of the preceding discussion, an integrated model that combines VBN and TPB is proposed, incorporating variables such as self-identity, perceived policy effectiveness, and school education for analysis. Figure 1, as shown above, illustrates this integrated model, which presents research hypotheses aimed at explaining college students’ intention to engage in clothing and textiles recycling.

## 3 Methods

### 3.1 Measurement items

Data gathering for this research was conducted through a questionnaire-based survey comprising two sections. The fundamental demographic information from the participants, encompassing gender, age, education level, and clothing expenditure, was gathered in the first section. The second section consisted of scale items designed to assess the factors that influence the involvement of college students in clothing and textiles recycling. The respondents assessed their level of agreement on a 7-point Likert scale. The scale items were selected from recognized scales in existing literature in order to prove the questionnaire’s validity and reliability.

Specifically, items for biospheric values and altruistic values were drawn from research conducted by Sanchez-Garcia et al. (2021), Lee and Jan (2018), Kim et al. (2022), and Denley et al. (2020). Items for attitude, subjective norms, behavioral intention and perceived behavioral control were drawn from research conducted by Conradie et al. (2023), Khan et al. (2020), Chu and Chen (2016), and Nie et al. (2020). Items for self-identity were drawn from research conducted by Bai et al. (2021). Items for perceived policy effectiveness were drawn from research conducted by Shen et al. (2022) and Liu et al. (2017). Lastly, items for school education were drawn from research conducted by Alvarez-Risco et al. (2021) and Truong et al. (2022).

To address any potential shortcomings in the questionnaire design, three professors were invited to conduct a pre-test of the questionnaire content. This was done to ensure that the scale items effectively measured college students’ behavioral intentions in participating in clothing and textiles recycling. Subsequently, ten college students were invited to complete the questionnaire, and their feedback on any issues encountered during the questionnaire completion process, such as ambiguous or difficult-to-understand items, was recorded. Further refinements to the verbiage and formulation of the questionnaire items were carried out based on feedback provided by both experts and participants. Table 1 presents all the scale items.

**Table 1:** Questionnaire items.

Constructs	Items	Reference(s)
Biospheric values (BV)	BV1: I should protect nature and environment.	Sanchez-Garcia et al. (2021), Lee and Jan (2018)
	BV2: I am an inseparable part of nature.	
	BV3: I respect the earth.	
	BV4: I should share my efforts in building a beautiful world.	
Altruistic values (AV)	AV1: Men were born equal.	Kim et al. (2022), Denley et al. (2020)
	AV2: Peace should prevail in the world.	
	AV3: Society should be ruled by justice.	
	AV4: I should be an altruist.	
Attitude (ATT)	ATT1:It is good to participate in clothing and textiles recycling.	Conradie et al. (2023), Khan et al. (2020)
	ATT2: It is wise to participate in clothing and textiles recycling.	
	ATT3: It is useful to participate in clothing and textiles recycling.	
	ATT4: It is beneficial to participate in clothing and textiles recycling.	
Subjective norm (SN)	SN1: My teachers support me to participate in clothing and textiles recycling.	Chu and Chen (2016), Nie et al. (2020)
	SN2: My family support me to participate in clothing and textiles recycling.	
	SN3: My classmates support me to participate in clothing and textiles recycling.	
	SN4: The mass media support me to participate in clothing and textiles recycling.	
Perceived behavioral control (PBC)	PBC1: It is on my own will to participate in clothing and textiles recycling.	Chu and Chen (2016), Nie et al. (2020)
	PBC2: I possess the knowledge and ability to participate in clothing and textiles recycling.	
	PBC3: I have the opportunity and resources to participate in clothing and textiles recycling.	
	PBC4: I can participate in clothing and textiles recycling without any help	
Behavioral intention (BI)	BI1: I intend to participate in clothing and textiles recycling in the future.	Chu and Chen (2016), Nie et al. (2020)
	BI2: I hope that I will participate in clothing and textiles recycling.	
	BI3:I will recommend clothing and textiles recycling to others.	
	BI4: I will post my unused old clothing on recycling platforms.	
Self-identity (SI)	SI1: I consider myself as an environmentalist.	Bai et al. (2021)
	SI2:I think I care about environmental issues.	
	SI3: I think I will be proud to live in an environment friendly way.	
	SI4: I am willing to participating in sustainable recycling.	
Perceived policy effectiveness (PPE)	PPE1: The government has encouraged the clothing and textiles recycling.	Shen et al. (2022), Liu et al. (2017)
	PPE2: The government has provided clothing and textiles recycling bins and door-to-door service.	
	PPE3:The government policies facilitate my participation to clothing and textiles recycling.	
	PPE4: The government policies have raised public concerns for environmental protection.	
School education (SE)	SE1: My school has provided relevant environmental courses.	Alvarez-Risco et al. (2021), Truong et al. (2022)
	SE2: My school has organized environmental lectures and clothing and textiles recycling activities.	
	SE3: My school has arranged environmental seminars or conferences.	
	SE4: My school have taught me to live in an environmental friendly way.	

### 3.2 Data collection

The data acquisition for this investigation occurred during the period spanning from June to August 2023, utilizing a combination of field surveys and online surveys. The survey sampling method involved stratified sampling and random sampling. Considering the large scale and representativeness of the college student group in the Eastern China region, the field surveys were conducted in regions including Jiangsu Province, Shandong Province, and Zhejiang Province. In each province, three typical cities were chosen, including three new first-tier cities (Nanjing, Qingdao, and Hangzhou), three second-tier cities (Wuxi, Jinan, and Wenzhou), and three third-tier cities (Zhenjiang, Dezhou, and Huzhou). In each city, 40–60 college students were randomly selected, resulting in 452 completed questionnaires, covering 26 universities. Additionally, questionnaires were distributed through social media platforms, with data primarily from the Eastern China region, including Jiangsu Province, Zhejiang Province, Fujian Province, and Shanghai Municipality. A total of 265, 198, 146, and 179 questionnaires were collected from these areas, respectively, amounting to 910 questionnaires collected online. Overall, 1,362 questionnaires were collected. To ensure data quality, specific questions were included in the questionnaire to filter out invalid responses, such as instructing respondents to select a specific option. Furthermore, questionnaires with a completion time of less than two minutes were excluded from the analysis. After applying these criteria, a sum of 1,027 valid questionnaires was obtained, yielding an effective response rate of 75.4%.

### 3.3 Data analysis method

SEM is a quantitative technique used to represent, measure, and analyze complex causal relationships among sample data through model equations. It is considered the optimal method for estimating data models (Little and Kline, 2016). SEM consists of two key components: the measurement model and the structural model. The measurement model analyses the associations between latent variables and observed variables, and the structural model establishes connections between latent variables and examines the paths (Byrne, 2013).

For the current study, SPSS software was used to analyze demographic data, assess scale reliability and validity, and examine moderation effects. The AMOS software was utilized for confirmatory factor analysis (CFA) and SEM analysis (Gkargkavouzi et al., 2019). Firstly, CFA was conducted to evaluate the convergent and discriminant validity of the measurement model (Anderson and Gerbing, 1988). Secondly, an evaluation of the global fit of the SEM was conducted (Hu and Bentler, 1999). Finally, SEM was employed for the analysis of the postulated pathways and causal relationships, and the PROCESS analysis was utilized to examine moderation variables (Sun et al., 2014).

## 4 Results

### 4.1 Respondent profile

According to the data presented in Table 2, the sample exhibits a nearly equal proportion of males and females. In relation to education level, the respondents predominantly consist of undergraduate and postgraduate (master’s and above) students, with undergraduates accounting for 42.3% and postgraduates comprising 35.4% of the sample. The majority of respondents fall between the ages of 18 to 25. When considering the average monthly expenditure on clothing, 53.9% of the respondents spend between 0 and 500 yuan, 22.3% spend between 501 and 1,000 yuan, and 23.8% spend more than 1,000 yuan. Furthermore, it is noteworthy that 62.2% of the respondents have no former experience in clothing and textiles recycling.

**Table 2:** Respondent profile.

Variables	Specifications	Counts	Percentage (%)
Gender	Male	497	48.4
	Female	530	51.6
Age	18–22 years old	418	40.7
	23–25 years old	324	31.6
	26–30 years old	182	17.7
	>30 years old	103	10.0
Education level	Junior College	229	22.3
	Undergraduates	434	42.3
	Postgraduates and above	364	35.4
Monthly clothing expenditure	0–500 RMB	554	53.9
	501–1,000 RMB	229	22.3
	1,001–2,000 RMB	81	7.9
	2,001–3,000 RMB	80	7.8
	>3,000 RMB	83	8.1
Any clothing and textiles recycling experiences	Yes	388	37.8
	No	639	62.2

### 4.2 Measurement model analysis

SPSS was employed to assess the reliability and validity of the scales prior to conducting confirmatory factor analysis (CFA). The Cronbach’s alpha coefficients for the scales exhibited a range between 0.862 and 0.888, all exceeding the 0.8 threshold, signifying that the observed variables are reliable and consistent within each dimension. Moreover, the Kaiser-Meyer-Olkin (KMO) coefficient was calculated to evaluate the overall validity of the scale, yielding a value of 0.864, significantly higher than the recommended threshold of 0.5, thus denoting good overall validity of the scale (Hair et al., 2012).

Subsequently, CFA was conducted employing the AMOS software. All factor loadings fell within the range of 0.765 to 0.854, as presented in Table 3, which was higher than the suggested limit of 0.7, indicating good measurement reliability. All CR values, ranging from 0.862 to 0.888, exceeded the 0.7 threshold, indicating good internal consistency and reliability. Additionally, the average variance extracted (AVE) values fell within the range of 0.610 to 0.666, all surpassing the 0.5 threshold, demonstrating good convergent validity of the measurement model (Bagozzi and Yi, 1988). Discriminant validity, which assesses whether different constructs are statistically distinct, was evaluated as shown in Table 4. The square roots of the AVE values for each variable were compared to the standardized correlation coefficients outside the diagonal. The measurement model’s discriminant validity was confirmed as the square roots of the AVE values surpassed the corresponding correlation coefficients (Hair et al., 2017).

**Table 3:** Results of internal and convergent reliabilities.

Constructs	Items	Loadings	α	CR	AVE
Altruistic values (AV)	AV1	0.779	0.876	0.873	0.632
	AV2	0.782			
	AV3	0.808			
	AV4	0.811			
Biospheric values (BV)	BV1	0.765	0.888	0.875	0.638
	BV2	0.833			
	BV3	0.782			
	BV4	0.812			
Self-identity (SI)	SI1	0.814	0.886	0.874	0.633
	SI2	0.779			
	SI3	0.798			
	SI4	0.792			
Perceived behavioral control (PBC)	PBC1	0.796	0.877	0.877	0.641
	PBC2	0.801			
	PBC3	0.815			
	PBC4	0.791			
Attitude (ATT)	ATT1	0.809	0.862	0.881	0.649
	ATT2	0.811			
	ATT3	0.814			
	ATT4	0.787			
Subjective norm (SN)	SN1	0.780	0.874	0.886	0.660
	SN2	0.805			
	SN3	0.827			
	SN4	0.836			
School education (SE)	SE1	0.781	0.881	0.862	0.610
	SE2	0.768			
	SE3	0.778			
	SE4	0.797			
Perceived policy effectiveness (PPE)	PPE1	0.767	0.873	0.877	0.640
	PPE2	0.811			
	PPE3	0.811			
	PPE4	0.810			
Behavioral intention (BI)	BI1	0.793	0.875	0.888	0.666
	BI2	0.812			
	BI3	0.804			
	BI4	0.854			

**Table 4:** Results of discriminant reliability.

Constructs	1	2	3	4	5	6	7	8	9
Biospheric values	0.798								
Altruistic values	0.219	0.795							
Attitude	0.168	0.148	0.805						
Subjective norm	0.113	0.099	0.188	0.812					
Perceived behavioral control	0.230	0.173	0.093	0.055	0.801				
Self-identity	0.093	0.025	0.153	0.009	0.157	0.796			
School education	0.209	0.101	−0.108	−0.232	0.054	0.056	0.781		
Perceived policy effectiveness	0.240	0.053	−0.148	−0.235	0.093	0.002	0.533	0.800	
Behavioral intention	0.263	0.217	0.238	0.160	0.238	0.178	0.199	0.132	0.816

Lastly, to ensure the adequacy of the SEM, it is essential to assess the fit of the measurement model. Therefore, conducting an analysis and evaluation of the model fit is necessary (Segars, 1997). In order to optimize the fit of the measurement model, items with high chi-square values were removed, resulting in a modified model that meets the fit criteria. The final model exhibited a *χ*^2^ value of 635, a df value of 558, and a *χ*^2^/df value of 1.138, which falls below the suggested threshold of 3. Additionally, the GFI, AGFI, TLI, and CFI values were 0.967, 0.961, 0.996, and 0.996, respectively, all exceeding the threshold of 0.9. Furthermore, the RMSEA value was 0.012, which falls below the suggested threshold of 0.08. These fit indices meet established research standards, signifying a favorable fit of the model (Rönkkö and Cho, 2022).

### 4.3 Structural model analysis

Based on the established measurement model, the researchers examined the direct effects of behavioral intentions. The findings illustrated in Table 5 reveal that both altruistic values (β = 0.125, *p* < 0.001) and biospheric values (β = 0.158, *p* < 0.001) exert a significant positive influence on the intention to recycle used clothing. Therefore, hypotheses H1 and H2, which propose the positive relationship between these variables, are supported. Additionally, perceived behavioral control (β = 0.145, *p* < 0.001), attitude (β = 0.147, *p* < 0.001), and subjective norms (β = 0.100, *p* < 0.01) were discovered to exert significant positive impacts on behavioral intentions, thereby substantiating hypotheses H3, H4, and H5, respectively.

**Table 5:** Path coefficients of structural model and hypothesis testing (direct effect).

Structural path	Path coefficient	S.E.	C.R.	*p*-values	Hypothesis result
AV → BI	0.125	0.034	3.579	***	H1 supported
BV → BI	0.158	0.035	4.441	***	H2 supported
PBC → BI	0.145	0.035	4.133	***	H3 supported
ATT → BI	0.147	0.034	4.252	***	H4 supported
SN → BI	0.100	0.031	3.029	0.002	H5 supported
AV → PBC	0.129	0.035	3.597	***	—
AV → ATT	0.117	0.036	3.239	0.001	—
BV → PBC	0.190	0.036	5.254	***	—
BV → ATT	0.130	0.036	3.591	***	—
SI → PBC	0.136	0.034	3.909	***	—
SI → ATT	0.139	0.035	3.944	***	—
SI → SN	0.014	0.037	0.389	0.697	—

Moreover, the researchers employed the Bootstrap method to further investigate the mediating effects of perceived behavioral control, attitude, and subjective norms within TPB. A total of 5,000 bootstrap resampling iterations were conducted, establishing a 95% confidence interval for the analysis of mediating effects. The mediating effect’s significance was established by considering whether the confidence interval for the mediation test included zero or not (Streukens and Leroi-Werelds, 2016). The results of the mediation analysis, depicted in Table 6, reveal the subsequent findings. The first mediation chain, altruistic values → perceived behavioral control → behavioral intentions, exhibits a significant mediating effect, as the confidence interval does not include zero. This result endorses hypothesis H6a. Similarly, hypotheses H6b, H7a, H7b, H8a, and H8b are supported, as their respective mediation chains also demonstrate significant mediating effects. However, it is important to note that the confidence interval for the seventh mediation chain, self-identity → subjective norms → behavioral intentions, includes zero. Consequently, hypothesis H8c is not supported.

**Table 6:** Indirect effects on dependent variable BI.

Hypothesis path	Path coefficient	*p*-values	Confidence Intervals (95%)	Hypothesis result
AV → PBC → BI	0.018	0.001	0.008–0.035	H6a supported
AV → ATT → BI	0.017	0.001	0.007–0.034	H6b supported
BV → PBC → BI	0.027	0.001	0.013–0.048	H7a supported
BV → ATT → BI	0.019	0.001	0.007–0.036	H7b supported
SI → PBC → BI	0.019	0.001	0.008–0.038	H8a supported
SI → ATT → BI	0.020	0.001	0.009–0.041	H8b supported
SI → SN → BI	0.001	0.586	−0.006-0.010	H8c rejected

To study the moderating effects of school education and perceived policy effectiveness, regression analysis was conducted following the approach outlined by Hayes (2017). The outcomes presented in Table 7 signify the following findings. Firstly, there is no significant positive regulating effect of perceived policy effectiveness on the association between behavioral intentions and perceived behavioral control (β = 0.001, *p* > 0.1). Consequently, hypothesis H9a is not supported. However, perceived policy effectiveness has a significant positive regulating influence on the associations among attitude (β = 0.037, *p* < 0.05), subjective norms (β = 0.044, *p* < 0.05), and behavioral intentions, providing support for hypotheses H9b and H9c. This indicates that as perceived policy effectiveness increases, the positive influence of subjective norms and attitudes on the intention to recycle used clothing strengthens. Furthermore, school education demonstrates a significant positive moderating influence on the associations among attitude, subjective norms, and behavioral intentions, supporting hypotheses H10b and H10c. However, the positive moderating influence of school education on the connection between perceived behavioral control and behavioral intentions is not significant (β = 0.008, *p* > 0.1), consequently, hypothesis H10a is not substantiated.

**Table 7:** Moderating effect test.

Hypothesis path	Path coefficient	S.E.	*T*-values	*p*-values	Hypothesis result
PBC*PPE → BI	0.001	0.018	0.034	0.973	H9a rejected
ATT*PPE → BI	0.037	0.017	2.137	0.033	H9b supported
SN*PPE → BI	0.044	0.018	2.412	0.016	H9c supported
PBC*SE → BI	0.008	0.019	0.429	0.668	H10a rejected
ATT*SE → BI	0.077	0.019	4.104	0.000	H10b supported
SN*SE → BI	0.099	0.019	5.222	0.000	H10c supported

## 5 Discussion

The current study employed a comprehensive theoretical model to assess college students’ willingness to engage in clothing and textiles recycling behavior. A questionnaire survey was conducted to gather data, and an SEM was employed to examine the relationships between variables, leading to the following research discoveries:

The current research confirmed the significance of the theory TPB and VBN in understanding college students’ participation in clothing and textile recycling. Firstly, among the TPB variables, active attitude was found to be associated with a higher intention to participate in recycling, in line with prior research by Ru et al. (2019) and Sanchez-Garcia et al. (2021). Additionally, Pleeging et al. (2021) recognized attitude as a primary predictor of pro-environmental behavior. In this research, attitude exhibited the most substantial impact on college students’ willingness to participate in clothing and textiles recycling among the TPB variables, highlighting its importance in shaping behavior. Perceived behavioral control and subjective norms were also found to have significant direct impacts on the intention of students in colleges to participate in recycling, aligning with previous research that highlights their role as robust predictors of pro-environmental behavioral intentions (Yadav and Pathak, 2017; Zhang et al., 2020). Secondly, within the VBN variables, the research demonstrated the significance of biospheric and altruistic values in predicting college students’ intention to recycle used clothing, in alignment with previous research that underscores the impact of values on shaping behavior (Ojea and Loureiro, 2007; Lee and Jan, 2018; Sanchez-Garcia et al., 2021). Among the studied variables, biospheric values exhibited the most pronounced direct influence on the willingness of students in colleges to engage in recycling.

Regarding the indirect effects, the VBN variables (biospheric values and altruistic values) were found to exert indirect influences on college students’ behavioral intentions through the TPB variables (perceived behavioral control and attitude). This finding aligns with the results of Sanchez-Garcia et al. (2021) and Yadav and Pathak (2017), suggesting that the VBN variables serve as important antecedents to attitude and perceived behavioral control. Additionally, self-identity exerted a notable indirect impact on the intention of students in colleges to participate in recycling through the TPB, indicating that college students may develop a positive attitude toward recycling and a belief in their ability to take action, explaining their perception of the behavior. This result aligns with prior studies by Mancha and Yoder (2015) and Gkargkavouzi et al. (2019). However, self-identity did not exert an indirect influence on behavioral intentions via subjective norms, which is inconsistent with the findings of Gkargkavouzi et al. (2019). The primary reason for this discrepancy lies in differences in research contexts. The previous study focused on pro-environmental behavior in the private sphere with more emphasis on intrapersonal factors, whereas this study concentrates on the specific issue of clothing and textiles recycling and individuals pay more attention to the impact on others, thereby reducing the effectiveness of self-identity (Vlastelica et al., 2023). In terms of the indirect influence on college students’ willingness to engage in recycling, biospheric values had a stronger predictive power through perceived behavioral control.

Regarding the moderating effects on college students’ participation in clothing and textiles recycling, perceived policy effectiveness was found to enhance the influences of both subjective norms and attitudes on their intention to engage in this behavior, highlighting the significant role of policy factors in shaping college students’ willingness to participate. This discovery is in agreement with the outcomes obtained by Wan et al. (2014a). However, the moderating influence of perceived policy effectiveness on the relationship between perceived behavioral control and behavioral intentions was not significant, which is at variance with the findings of Wan et al. (2014a). The main reason for this inconsistency is the difference in the sample. The previous research selected Hong Kong citizens as the respondents who live in an environment where policies and resources for pro-environmental behaviors are relatively abundant. In contrast, the respondents in this study included college students from new first-tier, second-tier, and third-tier cities, where the facilities and policies for clothing and textiles recycling are not sufficient. Therefore, a significant moderating effect was not observed in this study (Li and Li, 2019; Mei, 2020). Similarly, school education was found to separately enhance the influences of attitude and subjective norms on college students’ intention to engage in clothing and textiles recycling. This suggests that the higher the quality of education provided by schools regarding clothing and textiles recycling, the more significant the influence of attitude and subjective norms on college students’ behavioral intentions. This discovery is congruent with the outcomes of Tseng et al. (2022). However, school education did not exhibit a moderating effect between perceived behavioral control and behavioral intentions. The discrepancy may be attributed to differences in research themes. The previous study concentrated on college student entrepreneurship, while education on clothing and textiles recycling might have been neglected in university curricula and there might also be a lack of convenient channels or facilities to facilitate student participation in recycling activities (Truong et al., 2022).

### 5.1 Theoretical implications

While some studies have integrated the VBN theory and the TPB in the context of pro-environmental behavior, these integrated models have primarily focused on psychological factors and have not adequately considered external factors such as policies and situational factors (Karimi and Mohammadimehr, 2022; Wang et al., 2022; Woosnam et al., 2022). Therefore, the current study proposed a comprehensive theoretical framework that incorporates three additional variables: self-identity, perceived policy effectiveness, and school education, building upon the foundations of VBN and TPB. The analysis of the model results provided a more robust explanation for individual behavioral intentions and offered a holistic approach, contributing to future research on similar pro-environmental behaviors. Moreover, it enhanced the understanding of the interrelationships between TPB and VBN variables.

While the VBN theory has primarily found application in the domains of ecotourism and energy conservation (Lee and Jan, 2018; Venugopal and Shukla, 2019; Denley et al., 2020; Al Mamun et al., 2022), its application in the context of clothing and textiles recycling has been limited. Additionally, existing research on clothing and textiles recycling has predominantly focused on community residents, with less attention given to college students as a distinct group. Furthermore, the moderating influences of school education and perceived policy effectiveness have only been explored within the spheres of entrepreneurial intentions and the practice of waste sorting (Shen et al., 2022; Tseng et al., 2022). Therefore, the present study applied VBN in examining clothing and textiles recycling and confirms the moderating roles of school education and perceived policy effectiveness on the intention of college students to engage in reusing, thus expanding the research in this field.

Furthermore, the findings of the current study suggest that biospheric values are the primary driving factor for predicting college students’ willingness to participate in clothing and textiles recycling, which diverges from previous research that highlighted the significance of individual attitudes (Rausch and Kopplin, 2021; Shrivastava et al., 2021). This variation may be ascribed to the high educational attainment of college students, leading to a stronger inclination toward biospheric values. This research furnished empirical substantiation for understanding the behavioral intentions of highly educated individuals.

### 5.2 Practical implications

Based on the empirical analysis results mentioned above, the authors proposed the following practical implications to enhance college students’ willingness to participate in clothing and textiles recycling:

In the TPB model, attitude and perceived behavioral control have the most significant and highest path coefficients in influencing the intention to engage in clothing and textiles recycling. Therefore, practical suggestions can be made from these two aspects. Firstly, universities should use a blended approach of online and offline education tailored to college students. Online social platforms can be utilized to disseminate knowledge about clothing and textiles recycling, while offline activities such as lectures, courses, and extracurricular events can be organized to cultivate students’ behavioral attitudes (Grieve et al., 2016). Secondly, enhancing college students’ willingness to participate in clothing and textiles recycling is crucial. One strategy for achieving this is by strategically arranging the layout and quantity of used clothing collection bins on campus. Additionally, providing doorstep collection services can significantly improve the convenience of participation. These actions can strengthen students’ perceived behavioral control and, as a result, increase their engagement in recycling efforts (Sandin and Peters, 2018).

As previously confirmed, VBN (biospheric and altruistic values) serves as an important antecedent to attitude and perceived behavioral control, exhibiting a significant direct influence on college students’ intention to engage in clothing and textiles recycling. Therefore, universities should strengthen the education of students’ values by using multimedia platforms to showcase documentaries related to environmental issues, enabling students to deeply understand the importance of clothing and textiles recycling and raise their sense of environmental crisis, thereby enhancing their willingness to participate (Xiong et al., 2013). Additionally, schools and families should encourage students to care about social welfare. School administrations can organize volunteer services, recognize and reward students’ participation through scholarships or social activity honors, and help students recognize the crucial impact of donating used clothing on people in impoverished areas. Parents should act as role models in their own behaviors to cultivate their children’s environmental and altruistic values (Li et al., 2022).

Self-identity, as an antecedent to attitude and perceived behavioral control, indirectly influences the intention to engage in clothing and textiles recycling. Therefore, community agencies and school administrations can involve college students in the decision-making process of used clothing recycling activities, such as determining the content and strategies of recycling initiatives, to make them feel closely connected to the behavior. Moreover, school administrations can organize engaging and meaningful used clothing recycling activities during the education and promotion process to enhance students’ sense of self-identity and enjoyment (Van der Werff et al., 2013).

Considering the path coefficient findings, the influence of school education as a moderating variable on the association between attitude, subjective norms, and behavioral intention is more significant than the perceived effectiveness of policies. Hence, schools play a crucial role in facilitating college students’ participation in clothing and textiles recycling. In addition to the aforementioned practical implications, schools should establish dedicated sustainability research departments to facilitate collaboration among universities in sustainable research and jointly undertake the mission of sustainable development (Leal Filho et al., 2018). Furthermore, schools should implement and monitor clothing and textiles recycling efforts by designating coordinators to be responsible for relevant tasks, with a focus on evaluating the effectiveness of implementation. They can establish an “ecological indicator management system,” construct an ecological monitoring platform, and provide services such as clothing and textiles recycling data, recycling footprints, and low-carbon ambassadors. Leveraging the power of social influence, schools can guide college students’ behavior in clothing and textiles recycling (Zhou et al., 2021). The perceived effectiveness of policies by college students also exerts a moderating effect on the association between attitude, subjective norms, and behavioral intention. Hence, the government should strengthen the regulation and management of the clothing and textiles recycling market, including provisions for recycling process, environmental standards, and safety norms, to ensure the legality and sustainability of recycling and create a favorable market environment for students’ participation in recycling activities (Boschmeier et al., 2023). Additionally, the government could adopt incentive measures, such as reward systems, tax benefits, or subsidies, to foster an atmosphere of society-wide participation in clothing and textiles recycling, thereby positively influencing the participation of college students (Wang et al., 2020). Lastly, the government can establish collaborations with the academic community, through which the latter could offer professional research support and technical guidance to help the government formulate more sound policies and management measures for clothing and textiles recycling and provide college students with more knowledge related to clothing and textiles recycling (Shen et al., 2021). Through the implementation of these policies and regulations, college students would have a stronger awareness of the necessity and usefulness of engaging in the recycling of clothing and textiles.

### 5.3 Limitations and further suggestions

While the present study has contributed, there remain certain constraints that require consideration in future research. To begin with, even though the questionnaire’s sample size is sufficient, the survey participants primarily include college students from Eastern China without considering subjects from other countries or other social groups, undermining the universality of the study’s results (Loyalka et al., 2012). Therefore, future research should broaden the scope of subjects to investigate the factors influencing individual participation in clothing and textiles recycling in a more objective and comprehensive fashion.

Secondly, relevant research has indicated that even amid individuals possessing a heightened environmental awareness, there are objective limiting factors that influence their behavioral intentions (Gifford and Chen, 2017). The external factors explored in this study are limited, especially in the context of the COVID-19 pandemic, without fully considering the potential impacts of perceived risks and economic and social factors on individual behavioral intentions. Thus, future research should thoroughly take into account external factors and individual differences in age, gender, and education level, to enhance the external validity of the research.

Lastly, this study solely relied on questionnaire surveys to collect research data, resulting in a certain degree of subjectivity in the findings. Future research could consider using relevant experimental methods for follow-up surveys to ensure reliability and objectivity of the research results.

## Data availability statement

The original contributions presented in the study are included in the article/supplementary material, further inquiries can be directed to the corresponding author.

## Ethics statement

Ethical review and approval was not required for the study on human participants in accordance with the local legislation and institutional requirements. Written informed consent from the patients/ participants or patients/participants’ legal guardian/next of kin was not required to participate in this study in accordance with the national legislation and the institutional requirements.

## Author contributions

NS: Formal analysis, Supervision, Writing – review & editing. DL: Data curation, Investigation, Methodology, Software, Writing – original draft, Writing – review & editing. JZ: Investigation, Resources, Writing – review & editing.
